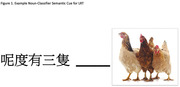# Noun‐Classifier as a Semantic Cue for Lexical Retrieval Therapy for svPPA patients: Treatment Evidence Supported by a Single‐case, Multiple Baseline Analysis

**DOI:** 10.1002/alz.088704

**Published:** 2025-01-09

**Authors:** Lorinda Li‐Ying Kwan Chen

**Affiliations:** ^1^ The Education University of Hong Kong, Tai Po, New Territories Hong Kong

## Abstract

**Background:**

Lexical retrieval therapy (LRT) has been proven to be an effective speech therapy for individuals with semantic variant primary progressive aphasia (svPPA) and semantic cue plays an important ingredient in LRT. In recent findings, differential performance in using and choosing noun‐classifiers amongst Chinese individuals with the three subtypes of PPA were observed. The current study aims to explore the treatment effect of employing noun‐classifier as a semantic cue of LRT for Cantonese‐speaking svPPA.

**Method:**

A single‐case, multiple‐baseline intervention study, modified lexical retrieval therapy using noun classifiers was conducted on two native Cantonese speakers (one male aged 74 and one female aged 76) diagnosed with svPPA. Eighteen sessions with daily home practice were conducted. Treatment outcomes were measured by naming accuracy and were measured at timepoints of pre‐treatment, immediate post‐treatment, and an additional 4 to 6 weeks post‐treatment (follow‐up stage). Primary and secondary outcome measures were the gain in accuracy of the naming of trained and untrained stimuli after therapy and at the follow‐up stage. One‐way ANOVA repeated measure was performed to demonstrate changes in treatment outcomes across time.

**Result:**

Both participants showed significant improvement (F(2, 142) = 11.48, p = .000 and F(1.60, 113.39) = 25.07, p = .000 for the two participants respectively) in naming trained stimuli in immediate post‐treatment with a large effect size (η^2^ = .14 and .26 for male and female participant respectively). Generalization to untrained stimuli was shown in immediate post‐treatment measurement (F(1.47, 104.28) = 12.91, p = .000 and F(1.51, 107.38) = 35.18, p = .000 for the two participants respectively) also with a large effect size (η^2^ = .15 and .33 for the two participants respectively). Maintenance over six weeks after treatment was recorded for both trained and untrained stimuli for both participants. There was statistically significant improvement in naming accuracy for trained stimuli (F(1.27, 90.31) = 15.58, p = .000 for male participant and F(1.23, 87.04) = 37.95, p = .000 for female participant) in 4‐week and 6‐week follow‐up as compared to pre‐treatment with large effect size (η^2^ = .18 and .35 for the two participants respectively).

**Conclusion:**

This study provides evidence for using noun‐classifiers as semantic cues in LRT for Chinese svPPA.